# Supplementary material for: Urine-Derived Stem Cell-Secreted Klotho Plays a Crucial Role in the HK-2 Fibrosis Model by Inhibiting the TGF-β Signaling Pathway
Source: Int J Mol Sci. 2022 Apr 30;23(9):5012. doi: 10.3390/ijms23095012 (PMC9105028; doi:10.3390/ijms23095012)

Urine-derived Stem Cell-secreted Klotho Plays a Crucial Role in the HK-2 Fibrosis Model by Inhibiting the TGF-β Signaling Pathway

Sang-Heon Kim [1,2,†], Jeong-Ah Jin [1,†], Hyung Joon So [1], Sung Hoon Lee [1], Tae-Wook Kang [1], Jae-Ung Lee [1], Dae Eun Choi [3], Young-Kwon Seo [2,*] and Hong-Ki Lee [1,*]

Supplementary Figure S1: The Klotho expression levels of UDSCs from healthy donors (n=5) and CKD patients (n=5).

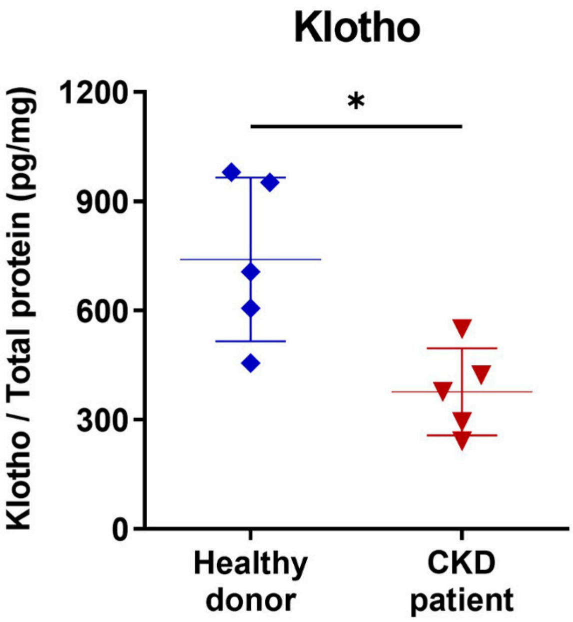

Supplementary Figure S2: The basal TGF-β1 secretion levels of various stem cells and HK-2 cells.

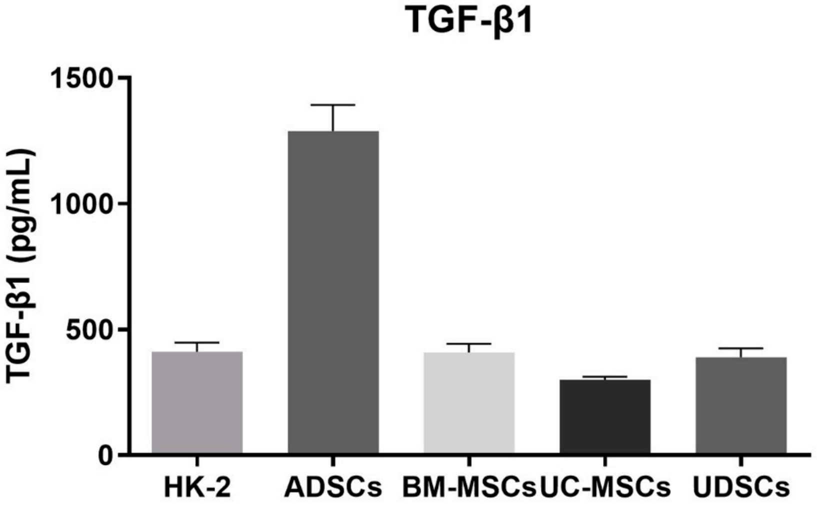

Supplementary Figure S3: The Klotho expression of UDSCs after TGF-β1 treatment for 72 hours.

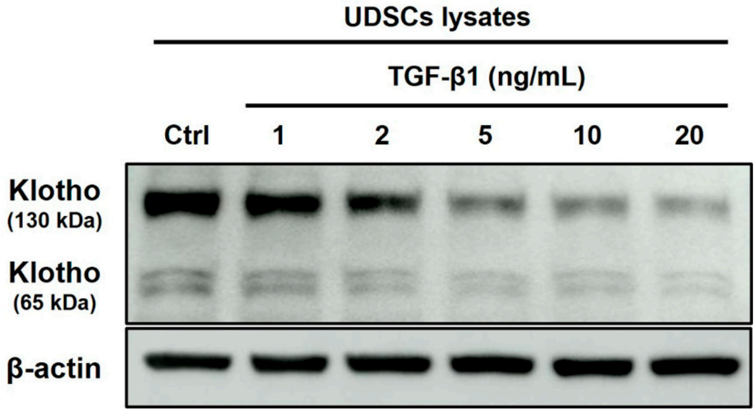

Supplement: Supplementary file 1 [file ijms-23-05012-s001.zip › Supplementary Figures - revision round 1.pdf]
