# Supplementary material for: Urine-Derived Stem Cell-Secreted Klotho Plays a Crucial Role in the HK-2 Fibrosis Model by Inhibiting the TGF-β Signaling Pathway
Source: Int J Mol Sci. 2022 Apr 30;23(9):5012. doi: 10.3390/ijms23095012 (PMC9105028; doi:10.3390/ijms23095012)
Supplement: Supplementary file 1 [file ijms-23-05012-s001.zip › Supplementary Tables - revision round 1.pdf]

Urine-derived Stem Cell-secreted Klotho Plays a Crucial Role in the HK-2 Fibrosis Model by Inhibiting the TGF-β Signaling Pathway

Sang-Heon Kim [1,2,†], Jeong-Ah Jin [1,†], Hyung Joon So [1], Sung Hoon Lee [1], Tae-Wook Kang [1], Jae-Ung Lee [1], Dae Eun Choi [3], Young-Kwon Seo [2,*] and Hong-Ki Lee [1,*]

Supplementary Table S1: Antibodies for flow cytometry

Analysis	Antibody	Manufacturer	Catalog no.
Flow cytometry	FITC Anti-human CD73	BD Biosciences	561254
	PE Anti-human CD90		555596
	FITC Anti-human CD105		561443
	PE Mouse Anti-Human CD133		566593
	FITC Anti-human SSEA4		560126
	PE Anti-human CD146		550315
	FITC Mouse Anti-human CD31		555445
	PE Anti-human CD34		555822
	FITC Anti-human CD45		555482

Supplementary Table S2: Primers for real-time PCR

Target Genes		Primer sequence	Product size (bp)	PCR cycles
E-Cadherin	F	CGAGAGCTACACGTTCACGG	20	45
	R	GGGTGTCGAGGGAAAAATAGG	21	45
N-Cadherin	F	TCAGGCGTCTGTAGAGGCTT	20	45
	R	ATGCACATCCTTCGATAAGACTG	23	45
Snail	F	TCGGAAGCCTAACTACAGCGA	21	45
	R	AGATGAGCATTGGCAGCGAG	20	45
Slug	F	CGAACTGGACACACATACAGTG	22	45
	R	CTGAGGATCTCTGGTTGTGGT	21	45
MMP-2	F	GATACCCCTTTGACGGTAAGGA	22	45
	R	CCTTCTCCCAAGGTCCATAGC	21	45
MMP-9	F	TGTACCGCTATGGTTACACTCG	22	45
	R	GGCAGGGACAGTTGCTTCT	19	45
Fibronectin	F	CTGGGATGCTCCTGCTGTCAC	21	45
	R	CTGTTTGATCTGGACCTGCAG	21	45
COL1A1	F	GAGGGCCAAGACGAAGACATC	21	45
	R	CAGATCACGTCATCGCACAAC	21	45

Supplementary Table S3: Antibodies for Western blot analysis

	Antibody	Manufacturer	Catalog no.
Western blotting	Anti-MMP-9 (D6O3H) Rabbit mAb	Cell Signaling Technology	13667
	Anti-α-Smooth Muscle Actin (D4K9N) Rabbit mAb		19245
	Anti-SMAD4 (D3R4N) Rabbit mAb		46535
	Anti-Phospho-Smad2 (Ser465/467)/Smad3 (Ser423/425) (D27F4) Rabbit mAb		8828
	Anti-Smad2/3 (D7G7) Rabbit mAb		8685
	Anti-E-Cadherin (24E10) Rabbit mAb		3195
	Anti-N-Cadherin (D4R1H) Rabbit mAb		13116
	Anti-TGF-β Antibody		3711
	Anti-Phospho-p44/42 MAPK (Erk1/2) (Thr202/Tyr204) (D13.14.4E) Rabbit mAb		4370
	Anti-p44/42 MAPK (Erk1/2) (137F5) Rabbit mAb		4695
	Anti-Ras (E4K9L) Rabbit mAb		91054
	Anti- Phospho-c-Raf (Ser338) (56A6) Rabbit mAb		9427
	Anti-c-Raf Antibody		9422
	Anti-Klotho mAb (Clone KM2076)	Cosmo bio	KO603
	Anti-β-Actin Antibody (C4)	Santacruz	sc47778
	Goat Anti-Rat IgG, (H+L), horseradish peroxidase conjugated	Invitrogen	31470
	Goat Anti-rabbit IgG, (H+L), horseradish peroxidase conjugated		31460
	Goat Anti-mouse IgG, (H+L), horseradish peroxidase conjugated		31430

Supplementary Table S4: Antibodies for immunofluorescence analysis

	Antibody	Manufacturer	Catalog no.
Immunofluorescence	Anti-Klotho (F-5)	Santacruz	sc515939
	Goat anti-Mouse IgG (H+L) Cross-Adsorbed Secondary Antibody, Alexa Fluor 488 (green)	Invitrogen	A-11001

Supplementary Table S5: List of abbreviations

Abbreviation	Full form
CKD	Chronic kidney disease
ESRD	End-stage renal disease
TIF	Tubulointerstitial fibrosis
EMT	Epithelial-mesenchymal transition
ECM	Extracellular matrix
IRI	Ischemia-reperfusion injury
HK-2	Human kidney-2
ADSCs	Adipose-derived stem cells
BM-MSCs	Bone marrow-derived stem cells
UC-MSCs	Umbilical cord-derived stem cells
UDSCs	Urine-derived stem cells
MSCs	Mesenchymal stem cells
TGF-β	Transforming growth factor-beta
MMP	Matrix metalloproteinase
α-SMA	Alpha-smooth muscle actin
SSEA4	Stage specific embryonic antigen 4
